# Extrinsic Apoptosis Pathway Altered by Glycogen Synthase Kinase-3*β* Inhibitor Influences the Net Drug Effect on NSC-34 Motor Neuron-Like Cell Survival

**DOI:** 10.1155/2017/4163839

**Published:** 2017-09-10

**Authors:** Jee-Eun Kim, Jung Hyun Lim, Gye Sun Jeon, Je-Young Shin, Suk-Won Ahn, Seung Hyun Kim, Kwang-Woo Lee, Yoon-Ho Hong, Jung-Joon Sung

**Affiliations:** ^1^Department of Neurology, Seoul Medical Center, Seoul, Republic of Korea; ^2^Department of Neurology, College of Medicine, Seoul National University, Seoul, Republic of Korea; ^3^Department of Neurology, College of Medicine, Chung-Ang University, Seoul, Republic of Korea; ^4^Department of Neurology, Institute of Biomedical Science, College of Medicine, Hanyang University, Seoul, Republic of Korea; ^5^Department of Neurology, Seoul National University College of Medicine, Seoul Metropolitan Government Boramae Medical Center, Seoul, Republic of Korea

## Abstract

Glycogen synthase kinase-3*β* (GSK-3*β*) inhibitors have been suggested as a core regulator of apoptosis and have been investigated as therapeutic agents for neurodegenerative diseases, including amyotrophic lateral sclerosis. However, GSK-3*β* has an interesting paradoxical effect of being proapoptotic during mitochondrial-mediated intrinsic apoptosis but antiapoptotic during death receptor-mediated extrinsic apoptosis. We assessed the effect of low to high doses of a GSK-3*β* inhibitor on survival and apoptosis of the NSC-34 motor neuron-like cell line after serum withdrawal. Then, we identified changes in extrinsic apoptosis markers, including Fas, Fas ligand, cleaved caspase-8, p38*α*, and the Fas-Daxx interaction. The GSK-3*β* inhibitor had an antiapoptotic effect at the low dose but was proapoptotic at the high dose. Proapoptotic effect at the high dose can be explained by increased signals in cleaved caspase-8 and the motor neuron-specific p38*α* and Fas-Daxx interaction. Our results suggest that GSK-3*β* inhibitor dose may determine the summation effect of the intrinsic and extrinsic apoptosis pathways. The extrinsic apoptosis pathway might be another therapeutic target for developing a potential GSK-3*β* inhibitor.

## 1. Introduction

Glycogen synthase kinase-3 (GSK-3) is a ubiquitous serine/threonine protein kinase that phosphorylates glycogen synthase and numerous other substrates. This implicates GSK-3 as a multifunctional modulator in critical cellular processes, including cell metabolism, gene expression, cell cycle division, development, and apoptosis [1, 2]. Dysregulation of GSK-3 plays an important role in the pathogenesis of various human diseases including psychiatric disorders, cancer, diabetics, inflammatory disease, and neurodegenerative diseases, including amyotrophic lateral sclerosis (ALS) [3–5]. GSK-3 usually exists in two isoforms, GKS-3*α* and GSK-3*β*, in mammals. Both isoforms are active in resting cells, and phosphorylation by serine-21 (GSK-3*α*) or serine-9 (GSK-3*β*) via the phosphatidylinositol 3-kinase (PI3K)/Akt pathway inhibits its activity [1]. Although GSK-3*α* and GSK-3*β* are structurally related, their functional activities are not identical [6]. The GSK-3*β* isoform is more abundant in the nervous system and has focused more attention on the involvement of GSK-3*β* in neurological diseases [7].

ALS is a catastrophic neurodegenerative disease that develops by progressive loss of motor neurons from the primary motor cortex to the anterior horn of the spinal cord. The pathological mechanism of ALS is unknown, but calcium or glutamate toxicity, abnormal protein aggregation, oxidative stress, immunity, or genetic defects have been proposed [8]. In addition, aberrant GSK-3*β* activity has been suggested as a potential etiology associated with neuronal apoptosis in ALS. Degenerating and normal-appearing motor neurons in the spinal cord of patients with sporadic ALS show upregulated GSK-3*β* expression [9]. A multi-immunoblotting proteomics study revealed elevated GSK-3*α* and GSK-3*β* activities in the thoracic cord of patients with sporadic ALS [10]. G93A and A4V mutant human Cu, Zn-superoxide dismutase (hSOD1) gene-transfected motor neurons consistently display GSK-3*β* hyperactivity along with inhibition of the PI3K/Akt pathway [11]. Taken together, these studies suggest the association of GSK-3*β* in ALS pathology.

GSK-3*β* inhibitors have received attention as new ALS therapeutic agents. The well-researched GSK-3*β* inhibitors valproate and lithium show significant neuroprotective effects in both* in vitro* and* in vivo* ALS studies. Valproate increases disease duration and prolongs survival of SOD1 mice [12]. Lithium treatment also improves motor function, delays disease progression, and decreases motor neuronal death in a dose-dependent manner in SOD1 transgenic mice [13, 14]. The neuroprotective effect was explained by the antiapoptotic effect of the GSK-3*β* inhibitor on the neurodegenerative disease.

However, GSK-3*β* is an intricate enzyme with contrasting effects on two classic apoptosis pathways [15]. GSK-3*β* promotes the mitochondrial-mediated intrinsic apoptosis pathway after cellular insult, whereas it suppresses the death receptor-mediated extrinsic apoptosis pathway. GSK-3*β* knockout mice reveal massive hepatocyte apoptosis; however, overexpression of GSK-3*β* also induces apoptosis [16, 17]. Although several studies have reported that lithium and other synthetic GSK-3*β* inhibitors have promising neuroprotective effects on many neurodegenerative diseases [17, 18], other studies have reported opposite results. Prostate cancer cell lines treated with lithium and another selective synthetic GSK-3*β* inhibitor enhance tumor necrosis factor-related apoptosis-inducing ligand (TRAIL)-induced apoptosis [19]. GSK-3*β* inhibitors enhance Fas-induced apoptosis in Jurkat cells and differentiated hippocampal neurons [20]. Previous studies are inconsistent regarding the effects of GSK-3*β* inhibitor on apoptosis.

These contradictory results of GSK-3*β* on the intrinsic and extrinsic apoptosis pathways seem to be environment- and cell-type-dependent [15, 21]. The extrinsic apoptosis pathway is initiated by the interaction between death receptor and its ligands. The death receptor expressing cells are categorized as either type I or type II cells based on whether apoptosis requires the activation of mitochondrial pathway [22]. Activated death receptors, such as Fas, recruit the Fas-associated death domain protein located in the cytoplasm and procaspase-8 to produce the death-inducing signaling complex (DISC). DISC passes the activation signal to caspase-8 and directly activates procaspase-3 to caspase-3 in type I cells, such as lymphocytes. The majority of cells (type II cells) follow the indirect, common intrinsic apoptosis signaling pathway. Active caspase-8 in type II cells cleaves Bid and interacts with mitochondria to release cytochrome C, which activated caspase-3 [15]. Interestingly, motor neurons follow a unique pathway and are regarded as type III cells [23]. Fas-triggered cell death in type III cells requires mutual activation of the classical caspase-8 and Daxx-p38-neuronal nitric oxide synthase (NOS) loop [23]. These motor neuron-specific extrinsic apoptosis pathways play an important function in death of motor neurons [23–25]. However, the influence of inhibiting GSK-3*β* on motor neurons, which are type III cells, has not been evaluated.

We hypothesized that there is an equilibrium point between reinforced extrinsic apoptosis and suppressed intrinsic apoptosis caused by GSK-3*β* inhibitors. We attempted to elucidate the net result of these two contrasting effects on death of motor neurons. The aims of this study were to investigate whether the extrinsic apoptosis pathway can be activated in motor neurons and to assess the change in the extrinsic pathway and its contribution to cell survival by inhibiting GSK-3*β* at varying degrees.

## 2. Materials and Methods

### 2.1. Reagents

GSK-3*β* inhibitor VIII, N-(4-methoxybenzyl)-N′-(5-nitro-1,3-thiazol-2-yl)urea, was purchased from Calbiochem (San Diego, CA, USA). Cell Counting Kit-8 (CCK-8) was from Dojindo (Tokyo, Japan). Mitochondria isolation kit for cultured cells and RIPA buffer were obtained from Thermo Scientific (Rockford, IL, USA). Annexin V and propidium iodide (PI) for flow cytometry were purchased from BD Pharmingen (San Diego, CA, USA). For the Western blot, the following specific antibodies were used: anti-tau (Invitrogen, Carlsbad, CA, USA), anti-phospho-tau (Ser396) (Invitrogen, Carlsbad, CA, USA), anti-Fas (Santa Cruz Biotech, Delaware, CA, USA), anti-Fas ligand (Santa Cruz Biotech, Delaware, CA, USA), anti-cleaved caspase-8 (Novus Biologicals, Littleton, CO, USA), anti-p38*α* (Santa Cruz Biotech, Delaware, CA, USA), anti-Daxx (Cell signaling, Beverly, MA, USA), anti-cleaved caspase-3 (Cell Signaling, Beverly, MA, USA), and anti-cytochrome C (Cell Signaling, Beverly, MA, USA).

### 2.2. Cell Culture, Serum Withdrawal, and GSK-3*β* Inhibitor Treatment

Mouse motor neuron-neuroblastoma hybrid cell line (NSC-34) (CELLutions Biosystems, Ontario, Canada) was maintained in Dulbecco's Modified Eagle's Medium (JBI, Korea) with 10% heat-inactivated fetal bovine serum (Gibco, Grand Island, NY, USA) and 1% penicillin-streptomycin (Gibco, Grand Island, NY, USA). Cells were grown in a humidified atmosphere of 5% CO_2_ and 95% O_2_ in a 37°C incubator. Serum deprivation was used to induce apoptosis in NSC-34 cells. Cells were plated at 1.5 × 10^4^/well in 96-well plates under normal conditions. The medium was replaced the next day with serum-deprived media. Cells were incubated for 72 hours (h) with serum withdrawal condition. Cell survival and apoptosis were each estimated at 0, 24, 48, and 72 h after serum deprivation by CCK-8 and flow cytometry, as described further within the text.

We selected a distinct serum deprivation time (60 h in our study) for further studies to compare the effects of the GSK-3*β* inhibitor at each dose. For drug treatment, cells were seeded at 1.5 × 10^4^/well in 96-well plates. The media was replaced the next day with serum-free media and/or GSK-3*β* inhibitor VIII (50, 200, and 1000 nM) and cells were incubated for 60 h.

### 2.3. Cell Viability Assay

CCK-8 uses highly water-soluble Dojindo's tetrazolium salt, WST-8 (2-(2-methoxy-4-nitrophenyl)-3-(4-nitrophenyl)-5-(2,4-disulfophenyl)-2H-tetrazolium, monosodium salt), which is known to be bioreduced by mitochondrial succinate dehydrogenases to yellow-colored formazan. Formed formazan is soluble in tissue media and the amount of formazan coloration is proportional to the number of viable cells. To assess cell viability, we used the CCK-8 assay as follows. NCS-34 cells were plated in 96-well plates at 1.5 × 10^4^/well. After 60 h of serum withdrawal conditions with or without GSK-3*β* inhibitor VIII, CCK-8 solution (10 *μ*l) was added in each well. Cells were incubated for 2 h at 37°C in a humidified atmosphere with 5% CO_2_ and 95% O_2_. Then, 96-well plates were measured at 450 nm absorbance using ELISA plate reader.

### 2.4. Flow Cytometry (Fluorescence-Activated Cell Sorting)

Annexin V is a phospholipid-binding protein that shows high affinity to phospholipid phosphatidylserine (PS) in the circumference with calcium (Ca2+). Normally, PS is located in the inner cytosolic leaflet. However, during apoptosis, PS redistributes into the outer leaflet of the plasma membrane and reacts with Annexin V-FITC. For flow cytometry, PI was concomitantly used with Annexin V-FITC as a standard viability probe. Because PI is unable to penetrate the cell membrane, only nucleic acids, DNA, and RNA of dead cells were stained with PI. Consequentially, early apoptotic cells were defined as Annexin V-FITC-positive and PI-negative groups. We estimated the early apoptotic cells by utilizing the Annexin V-FITC apoptosis detection kit following the prescribed protocols. Briefly, collected cells were washed twice with cold PBS and were resuspended in Annexin V binding buffer at 1 × 10^6^ cells/ml. We transferred 1 × 10^5^ cells/100 *μ*l to a 5 ml tube and added 5 *μ*l of Annexin V-FITC and 10 *μ*l PI. Cells were stained at room temperature in the dark for 15 min. After incubation, 400 *μ*l of Annexin V binding buffer was added to each tube which was analyzed by flow cytometry using CellQuest software within 1 h.

### 2.5. Western Blot Analysis

We evaluated the immunoreactivity of tau, phospho-tau, Fas, Fas ligand, cleaved form of caspase-8, p38*α*, cleaved form of caspase-3, and cytosolic cytochrome C by Western blot analysis. Briefly, cells were plated at a density of 2 × 10^6^ cells/100 mm dish and were cultured for 24 h in a humidified atmosphere of 95% O_2_-5% CO_2_ in a 37°C incubator. The media was replaced the next day with serum-free media and/or GSK-3*β* inhibitor (50, 200, and 1000 nM) and cells were incubated for 60 h. The cells were collected by pipetting, and cell pellets were washed twice in cold PBS. The cell pellets were incubated for 30 min on ice in radio immunoprecipitation assay (RIRA) buffer containing protease inhibitor and phosphatase inhibitor. Cell lysates were centrifuged for 15 min at 13000 rpm at 4°C and the resulting supernatants were used for the protein assay. To isolate the cytosolic cytochrome C from the mitochondrial cytochrome C, we applied mitochondria isolation kit following the enclosed manufacturer's instruction. Briefly, harvested cell suspensions were centrifuged at ~850 rpm for 2 min and 800 *μ*l of mitochondrial isolation agent A, 10 *μ*l of mitochondrial isolation reagent B, and 800 *μ*l of mitochondrial isolation reagent C were added in this order, with 5 sec intermittent mixing with each addition. Mixed solutions were shaken at a medium speed for 5 sec and placed on ice for 2 min after each mixture. After centrifugation at 700*g* for 10 min at 4°C, we transferred the supernatant to a 2 ml tube and subsequently performed centrifugation at 12,000 rpm for 15 min at 4°C. The resultant supernatant, the cytosolic fraction, was placed on a new tube and was used for immunoblotting of cytosol cytochrome C. The BioRad protein assay kit was used for detecting the protein concentrations of the lysates. For Western blotting, 50 *μ*g of the total protein from each sample was boiled for 5 min and was then left in ice. Then, each 50 *μ*g sample was electrophoresed on 4~12% sodium dodecyl sulfate (SDS) polyacrylamide gel and was transferred to a polyvinylidene difluoride membrane. The membrane was blocked for 1 h with 5% dry milk and was then incubated with primary antibody at 4°C overnight followed by washing with Tris-buffered saline containing 0.05% Tween-20 (TBST). Next, it was processed with secondary anti-mouse or anti-rabbit antibodies conjugated to horseradish peroxidase for ECL detection.

### 2.6. Immunoprecipitation

To observe the change of the interaction of Fas-Daxx after each treatment, immunoprecipitation (IP) was performed as previously described [26]. Treated cells were incubated with anti-Fas antibody at 4°C overnight. Protein A resin was added slowly to the antigen-antibody complex, which was then mixed for 2 h at room temperature. IP buffer (25 mM Tris, 150 mM NaCl, pH 7.2) was then added to the mixture, which was centrifuged for 2~3 min at 2500 rpm. Supernatants were discarded. To elute the immune complex, 50 *μ*l sample buffer was added to Sepharose beads, followed by boiling for 5 min at 95°C. The resultant samples of each group were electrophoresed on 4~12% SDS polyacrylamide gel and were transferred to a polyvinylidene difluoride membrane. Finally, we performed Western blot analysis using anti-Daxx antibody in the aforementioned manner.

### 2.7. Statistical Analysis

All the data related to viability, apoptosis measurement, and arbitrary units of Western blot analysis are presented as mean ± standard error (SE) from more than three or four independent tests. For the statistical comparison of the GSK-3*β* inhibitor's effect with that of control, we used Tukey's multiple comparison tests after one-way ANOVA (GraphPad Prism software). *p* < 0.05 was considered to be statistically significant.

## 3. Results

### 3.1. Effect of a GSK-3*β* Inhibitor on Cell Viability during Serum Deprivation

**Figure 1:**
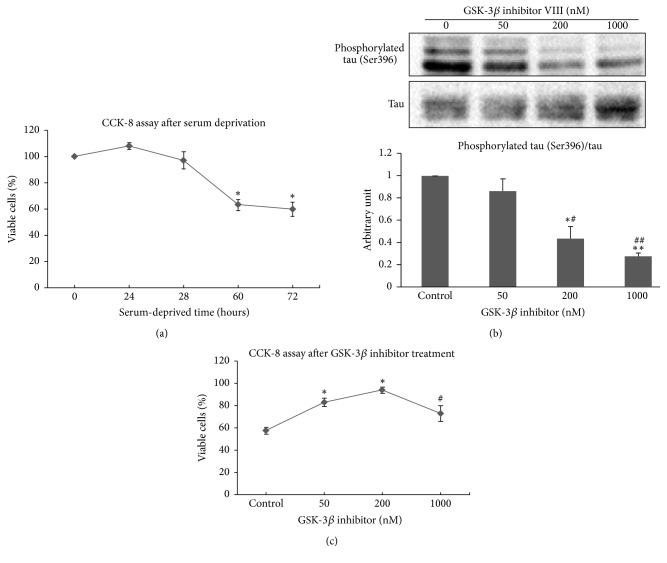
*Effect of the glycogen synthase kinase-3β (GSK-3β) inhibitor VIII on viability of serum-deprived NSC-34 cells*. (a) NSC-34 cell viability after serum deprivation was evaluated by the CCK-8 assay. NSC-34 cells were incubated for 72 h (h) under a serum withdrawal condition, and cell viability was measured with the CCK-8 assay. As serum deprivation time elapsed, cell viability decreased. Data are mean (% of viable cells of the control) ± standard error (SE). ^*∗*^*p* < 0.05 (compared with viability of control cells under normal conditions with growth factors). (b) GSK-3*β* activity was measured indirectly by measuring the immunoreactivity (IR) ratio of phosphorylated tau (Ser396)/total tau after GSK-3*β* inhibitor VIII treatment. NSC-34 cells were incubated in serum-deprived media with or without the GSK-3*β* inhibitor (0, 50, 200, and 1000 nM). Western blot results for phosphorylated tau (Ser396) and total tau are indicated following each concentration. As the GSK-3*β* inhibitor dose increased, the immunoreactivity (IR) ratio of phosphorylated tau (Ser396)/total tau decreased. Quantitative data of IR ratio is presented as arbitrary units. ^*∗*^*p* < 0.01 and ^*∗∗*^*p* < 0.001 (compared with control under serum deprivation only) and ^#^*p* < 0.05 and ^##^*p* < 0.01 (compared with groups treated with 50 nM GSK-3*β* inhibitor VIII). (c) CCK-8 assay after GSK-3*β* inhibitor treatment in 60 h serum-deprived NSC-34 cells. Cell viability at each GSK-3*β* inhibitor concentration is marked as mean (% of cell viability under normal conditions) ± SE. Protective effect was maximal at 200 nM of the GSK-3*β* inhibitor, but these protective effects decreased above 200 nM. ^*∗*^*p* < 0.05 (compared with control under serum deprivation only). ^#^*p* < 0.05 (compared with 200 nM GSK-3*β* inhibitor-treated group).

NSC-34 cells were incubated for 72 h under a serum withdrawal condition, and cell viability was determined using the CCK method at 0, 24, 48, 60, and 72 h after serum deprivation. As shown in Figure 1(a), cell viability decreased with serum deprivation time. Cell viability was 97.11 ± 6.45% after 48 h, 63.05 ± 8.24% after 60 h, and 59.95 ± 10.82% after 72 h of serum deprivation. We selected the 60 h serum deprivation condition for further studies, because cell viability was moderately decreased to 60–70% at the period that was thought to be sufficient to check neuroprotective effect of drug.

NSC-34 cells were treated with different doses of the GSK-3*β* inhibitor VIII (0, 50, 200, and 1000 nM) and were exposed to a serum-deprived condition for 60 h. The microtubule-associated protein tau, which is a GSK-3*β* substrate, was selected to evaluate GSK-3*β* activity. Because GSK-3*β* phosphorylates tau at many sites including the serine 396 residue, GSK-3*β* activity can be indirectly measured by the ratio of phosphorylated tau (Ser396) to total tau immunoreactivity [27]. The immunoreactivity ratio of phosphorylated tau (Ser396)/total tau decreased significantly as the concentration of GSK-3*β* inhibitor increased (Figure 1(b)). We confirmed that the GSK-3*β* inhibitor VIII was effective in NCS-34 cells and that this inhibitory action was dose-dependent.

We next then evaluated how NSC-34 cell viability would change according to the GSK-3*β* inhibitor concentration. NSC-34 cells showed significantly improved cell viability at 50 and 200 nM concentrations of the GSK-3*β* inhibitor (82.82 ± 3.77%, *p* < 0.01 at 50 nM cells; 93.88 ± 2.91%, *p* < 0.01 at 200 nM) compared to that in control (57.47 ± 3.04% survival), which was only serum-deprived. However, cell viability decreased significantly at 1000 nM of the GSK-3*β* inhibitor compared to that at 200 nM (93.88 ± 2.91% versus 72.89 ± 7.08%, resp., *p* < 0.05) (Figure 1(c)). Cell viability was maximal in the 200 nM GSK-3*β* inhibitor-treated cells, and viability decreased at greater concentrations.

### 3.2. High-Dose GSK-3*β* Inhibitor Treatment Reinforces Late Apoptosis during Serum Deprivation

**Figure 2:**
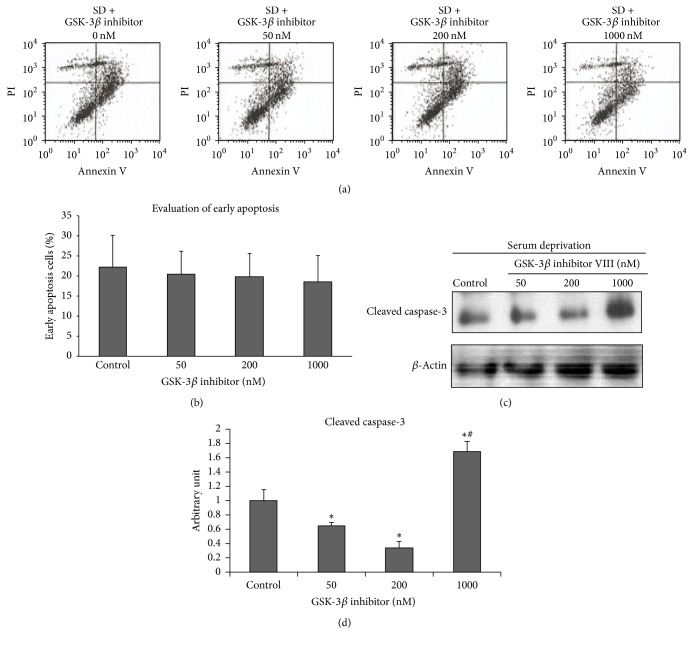
*Effect of the glycogen synthase kinase-3β (GSK-3β) inhibitor VIII on early/late apoptosis in NCS-34 cells under serum withdrawal conditions*. (a) NSC-34 cells were incubated in serum-deprived media with or without the GSK-3*β* inhibitor at the indicated doses for 60 h. Harvested cells were then stained with an Annexin V-FITC kit and were applied to a fluorescence-activated cell sorting analysis. Early apoptotic cells were Annexin V-positive (right lower). (b) Quantitative representation of cells in early apoptosis. No difference in the proportion of early apoptotic cells was detected among the four GSK-3*β* inhibitor VIII-treated groups. Presented as mean (% of all cells counted) ± standard error (SE). (c) NSC-34 cells in late apoptosis were indirectly assessed by detecting the change in cleaved caspase-3 signaling by Western blot analysis. Actin was used as the loading control. (d) Quantitative immunoreactivity data of cleaved caspase-3, expressed in arbitrary units and normalized to the control. Reduced cleaved caspase-3 signals were noted in the low-dose GSK-3*β* inhibitor VIII-treated group but the signal increased significantly in the 1000 nM GSK-3*β* inhibitor treatment. ^*∗*^*p* < 0.05 (compared with control under serum deprivation only). ^#^*p* < 0.05 (compared with 200 nM GSK-3*β* inhibitor-treated group).

We assessed two delegate apoptosis markers, flow cytometry after Annexin V and propidium iodide (PI) staining and changes in cleaved caspase-3, which is the active form of caspase-3, by Western blot analysis to determine whether the changes in viability in response to the GSK-3*β* inhibitor VIII treatment resulted from alterations in the apoptotic response. Early apoptotic cells can be detected by estimating Annexin V-positive/PI-negative cells. NSC-34 cells were treated with the GSK-3*β* inhibitor VIII at different doses under serum-deprived conditions. No significant change in early apoptosis was observed between the different GSK-3*β* inhibitor VIII doses based on the Annexin V-FITC assay results (Figures 2(a) and 2(b)). These insignificant differences might be influenced by the proportion of Annexin V-negative but caspase-3 active cells, which is also on their early apoptotic state. However, cleaved caspase-3 decreased significantly at low doses (50 and 200 nM) of the GSK-3*β* inhibitor VIII compared with that in the control (*p* < 0.05; *p* < 0.05). This decrease peaked at 200 nM. Then, cleaved caspase-3 increased significantly compared to that in the control and low-dose treated groups (*p* < 0.05) (Figures 2(c) and 2(d)).

### 3.3. Extrinsic Apoptosis Markers Increase with Increased GSK-3*β* Inhibitor Concentration during Serum Deprivation-Induced Neuronal Apoptosis

NSC-34 cells from each GSK-3*β* inhibitor concentration group were immunoblotted to determine how viability and apoptosis changed according to GSK-3*β* inhibitor concentration and whether changes in extrinsic apoptosis signals accounted for these changes. Fas, Fas ligand, cleaved caspase 8, and p38*α* were investigated as markers of the extrinsic apoptosis pathway, and cytochrome C was selected as a marker of the common apoptosis pathway.

**Figure 3:**
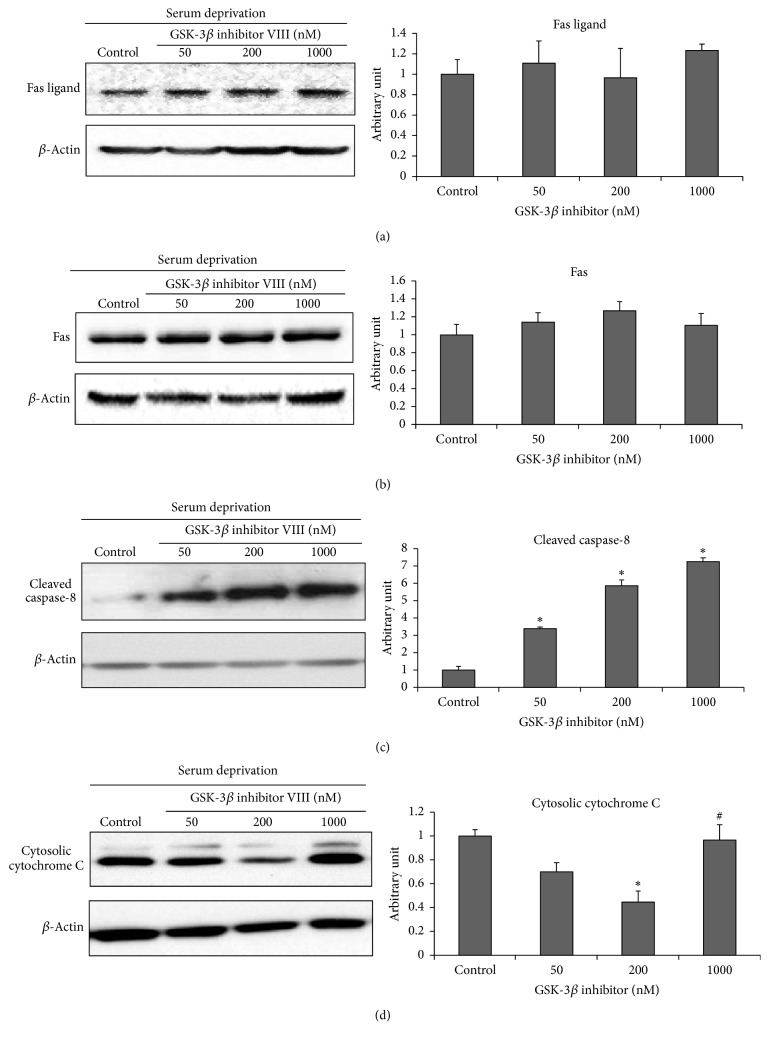
*Changes in the classical FADD-caspase-8 extrinsic apoptosis pathway markers after glycogen synthase kinase-3β (GSK-3β) inhibitor VIII treatment*. Changes in immunoreactivity (IR) of the classical extrinsic apoptosis markers based on the treated GSK-3*β* inhibitor concentration are presented in an enhanced chemiluminescence radiograph as quantitative values. ((a) and (b)) IR of Fas and the Fas ligand, which are the first step in the FADD-caspase-8 extrinsic pathway, did not change in the different GSK-3*β* inhibitor-treated groups. (c) IR of cleaved caspase-8 increased in a dose-dependent manner. (d) IR of cytosolic cytochrome C, a common apoptosis marker, decreased in the low-dose group and was minimized at 200 nM. The 1000 nM GSK-3*β* inhibitor VIII-treated cells showed a U-shaped increasing IR pattern. ^*∗*^*p* < 0.05 (compared with control under serum deprivation only). ^#^*p* < 0.05 (compared with 200 nM GSK-3*β* inhibitor-treated group).

**Figure 4:**
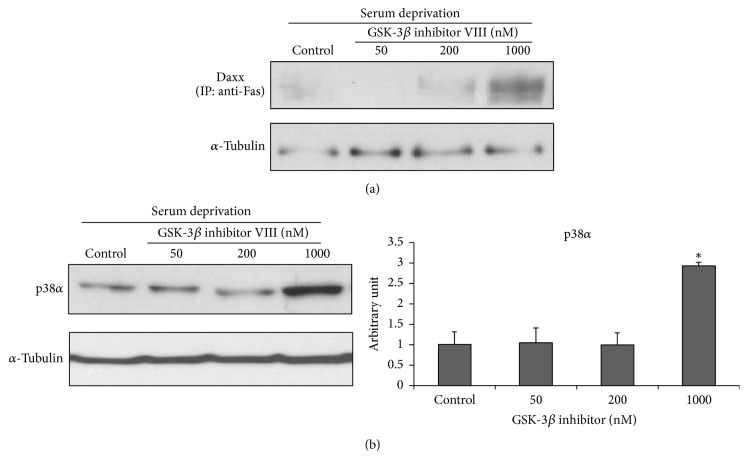
*The motor neuron-specific Daxx-p38α extrinsic apoptosis pathway is activated by glycogen synthase kinase-3β (GSK-3β) inhibitor VIII treatment*. (a) Proteins from each group were extracted and immunoprecipitated with anti-Fas antibody. The precipitates were subjected to sodium dodecyl sulfate polyacrylamide gel electrophoresis and Western blot analysis with anti-Daxx antibody. The Fas-Daxx interaction increased significantly in 1000 nM GSK-3*β* inhibitor-treated cells. (b) p38*α* immunoreactivity (IR) is presented in an enhanced chemiluminescence radiograph as quantitative values. Band signal intensity increased nearly threefold in 1000 nM GSK-3*β* inhibitor VIII-treated cells, compared with that in the control. No differences in IR were detected in the low-dose treated groups compared with the control group.

Treatment with the GSK-3*β* inhibitor VIII did not change Fas or Fas ligand immunoreactivity (IR) under serum-deprived conditions (Figures 3(a) and 3(b), resp.). However, cleaved caspase-8 IR increased after GSK-3*β* inhibitor VIII treatment in a dose-dependent manner compared with that in the control (serum-deprived only) (Figure 3(c)). The IR of the common apoptosis marker cytochrome C showed a similar change to that seen in cleaved caspase-3. The low-dose (50–200 nM) GSK-3*β* inhibitor VIII-treated cells showed decreased cytochrome C IR and IR was minimal at 200 nM compared with that of the control. The 1000 nM GSK-3*β* inhibitor VIII-treated cells showed an increasing IR pattern resulting in a U-shaped dose-response curve (Figure 3(d)). Changes in the motor neuron-specific extrinsic apoptosis pathway markers p38*α* and Daxx were analyzed. We carried out immunoprecipitation assays on the Fas-Daxx interaction to identify how GSK-3*β* activity changes. As a result, Fas-Daxx interactions increased significantly in the 1000 nM GSK-3*β* inhibitor VIII-treated group compared to that in the control (Figure 4(a)). The p38*α* band signal increased nearly threefold in the 1000 nM GSK-3*β* inhibitor VIII-treated cells, compared with that in the control (Figure 4(b); *p* < 0.05). In contrast, p38*α* expression in the low-dose treated groups did not change. These findings agree with the signal change observed during Fas-Daxx interactions.

## 4. Discussion

We demonstrated that a GSK-3*β* inhibitor affects apoptosis in NSC-34 cells, which have the characteristics of motor neurons. The antiapoptotic effect of the GSK-3*β* inhibitor observed at low doses was not observed at high doses, and the inhibitor seemed to be proapoptotic. These anti- and proapoptotic effects can be explained, in part, by the paradoxical effect of GSK-3*β* inhibition on the intrinsic and extrinsic apoptosis pathways and the shift of balance depending on the degree of enzyme inhibition. This notion is supported by our findings of altered extrinsic apoptosis components, including Fas, Fas ligand, caspase-8, p38, and the Fas-Daxx interaction.

**Figure 5:**
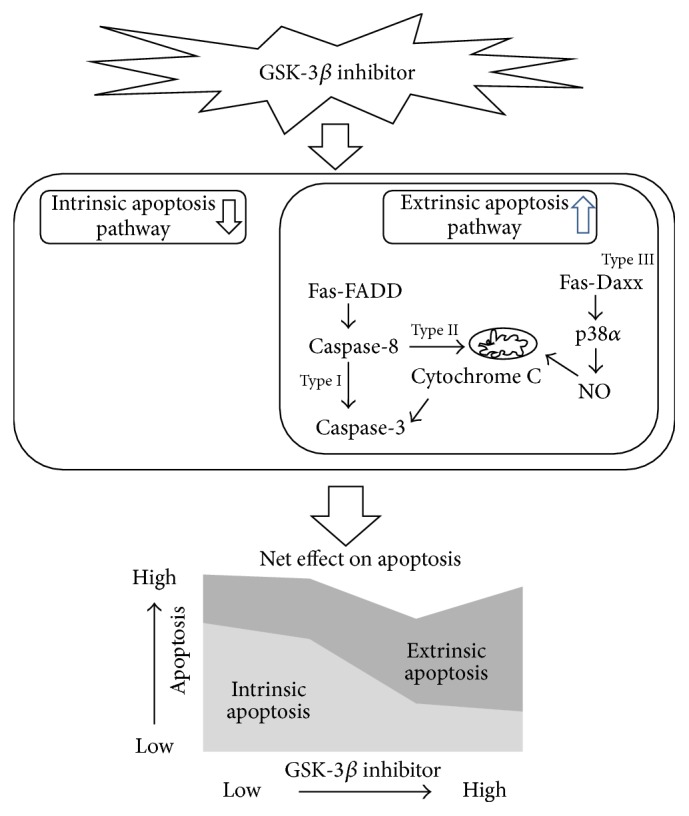
*Net effect of changes in intrinsic and extrinsic apoptosis caused by inhibiting glycogen synthase kinase-3β (GSK-3β) in motor neurons*. Illustration shows the change in the intrinsic and extrinsic apoptosis pathways affected by the GSK-3*β* inhibitor. The greater the decrease in GSK-3*β* activity was, the greater the decrease in intrinsic apoptosis was observed. However, extrinsic apoptosis increased paradoxically.

Different GSK-3*β* inhibitor doses did not affect the death receptor Fas or its ligand, and the Western blot changes in the Fas and Fas ligand did not mirror the strength of their interaction. However, cleaved caspase-8 expression increased in a dose-dependent manner. The relative IR ratio of p38*α* and the Fas-Daxx interaction increased significantly in the 1000 nM GSK-3*β* inhibitor treatment. These changes in the extrinsic markers according to GSK-3*β* inhibitor concentration may explain the viability and apoptosis assay results showing a U-shaped dose response to the GSK-3*β* inhibitor. This is an important point to discuss because we might miss a potential ALS therapeutic tool or target without understanding the changes in the interaction between extrinsic apoptosis and intrinsic apoptosis during GSK-3*β* inhibitor treatment in motor neurons. As we illustrated in Figure 5, the antiapoptotic effect from inhibiting intrinsic apoptosis with the GSK-3*β* inhibitor may have been reversed by the proapoptotic effect due to reinforcement of extrinsic apoptosis at the high concentration. The most interesting result was that the type III cell-specific extrinsic apoptotic markers, p38*α* and the Fas-Daxx interaction, did not increase significantly in response to low concentrations of the GSK-3*β* inhibitor, but Fas-Daxx interaction and p38*α* interaction signaling rose steeply at the high GSK-3*β* inhibitor concentration when a neuroprotective effect was no longer seen. These abrupt changes were not observed in common extrinsic apoptotic markers shared by type I and type II cells. These findings suggest that the Daxx-p38-neuronal NOS loop, which is a distinctive extrinsic apoptotic pathway of motor neuron, may play a more significant role in this phenomenon. This suggestion is supported by a previous finding that activation of Fas/NO feedback is essential for death of motor neurons. That study also showed reduced activation of the Fas/NO feedback loop in dominant negative Daxx SOD1 (G93A) transgenic mice [27]. They suggested that motor neurons may die after exceeding a threshold from a chronic insult, which would result in an amplified death signal [27]. Transgenic mice with the dominant negative form of GSK show rapid motor deficits and neuronal apoptosis. The finding that this neurotoxicity was reversed under a Fas-deficient background reinforces the importance of the Fas/NO feedback loop in motor neuron degeneration [28].

The paradoxical action of GSK-3*β* on apoptosis explains a phenomenon that was previously difficult to understand. Overexpression of GSK-3*β* or GSK-3*β* knockout mice both induce apoptosis [16, 17]. The effects of lithium and other novel synthetic GSK-3*β* inhibitors on apoptosis are contradictory [18, 29]. The recent failure of a large clinical trial to show the effectiveness of lithium treatment in patients with ALS may have been influenced by these complex actions of GSK-3*β* [30]. One study suggested that oxidative stress-induced cell death decreases after treatment with GSK-3*β* inhibitor II and GSK-3*β* inhibitor VIII at certain dose ranges, whereas higher dosages of the GSK-3*β* inhibitor promote apoptosis [31], which coincides well with our viability assay results. We also previously found reinforced extrinsic apoptosis signaling in an* in vivo* ALS model during GSK-3*β* inhibitor treatment [32].

In this study, we confirmed activation of the extrinsic apoptosis pathway during GSK-3*β* inhibitor treatment in NCS-34 motor neurons. Extrinsic apoptosis signaling was influenced by GSK-3*β* inhibitor concentration and some significant extrinsic apoptosis markers, as cleaved caspase-8 increased in a concentration-dependent manner. The Fas-Daxx interaction and p38*α* increased abruptly in response to the high dose of the GSK-3*β* inhibitor, suggesting a unique role in motor neuron degeneration. Cell death observed under serum-deprived conditions with the GSK-3*β* inhibitor showed an optimal dose range that maximized the neuroprotective effect, whereas the GSK-3*β* inhibitor may well be toxic above that dose. We suggest that there may be a balancing point between the inhibited intrinsic apoptosis and augmented extrinsic apoptosis effects caused by GSK-3*β* inhibitors, at which their summed effect is maximal for cell survival. GSK-3*β* inhibitors remain a potential new therapeutic drug for many neurodegenerative diseases, including ALS. However, investigators must consider both actions (intrinsic and extrinsic apoptosis pathways) of GSK-3*β* on apoptosis for clinical use and develop the most suitable and effective dose depending on whether the pro- or antiapoptotic effects of the GSK-3*β* inhibitor are sought. Another strategy would be to selectively block the extrinsic apoptosis pathway activated by a GSK-3*β* inhibitor [33].

## 5. Conclusions

Our results showed significant effects of a GSK-3*β* inhibitor on the extrinsic apoptosis pathway in motor neurons. Extrinsic apoptosis signaling was enhanced in motor neurons treated with a GSK-3*β* inhibitor, and intensity was strongly influenced by dose. The GSK-3*β* inhibitor's dose may determine the summation effect of the two apoptosis pathways.
